# Sympathetic neurons and chromaffin cells share a common progenitor in the neural crest *in vivo*

**DOI:** 10.1186/1749-8104-8-12

**Published:** 2013-06-18

**Authors:** Stella Shtukmaster, Marie Catherine Schier, Katrin Huber, Shlomo Krispin, Chaya Kalcheim, Klaus Unsicker

**Affiliations:** 1 Department of Molecular Embryology, Institute of Anatomy and Cell Biology University of Freiburg, Albertstr. 17, Freiburg D-79104, Germany; 2 Clinical Cooperation Unit Pediatric Oncology, German Cancer Research Center (DKFZ), Heidelberg D-69120, Germany; 3 Department of Medical Neurobiology, IMRIC and ELSC-Hebrew University of Jerusalem, Hadassah Medical School, POB 12272, Jerusalem 9112102, Israel

**Keywords:** Chicken embryo, Chromaffin cells, Neural crest, Single cell electroporation, Sympathetic neurons, Sympathoadrenal progenitors

## Abstract

**Background:**

The neural crest (NC) is a transient embryonic structure unique to vertebrates, which generates peripheral sensory and autonomic neurons, glia, neuroendocrine chromaffin and thyroid C-cells, melanocytes, and mesenchymal derivatives such as parts of the skull, heart, and meninges. The sympathoadrenal (SA) cell lineage is one major sub-lineage of the NC that gives rise to sympathetic neurons, chromaffin cells, and the intermediate small intensely fluorescent (SIF) cells. A key question is when during NC ontogeny do multipotent progenitors segregate into the different NC-derived lineages. Recent evidence suggested that sympathetic, sensory, and melanocyte progenitors delaminate from the thoracic neural tube (NT) in successive, largely non-overlapping waves and that at least certain NC progenitors are already fate-restricted within the NT. Whether sympathetic neurons and chromaffin cells, suggested by cell culture studies to share a common progenitor, are also fate segregated *in ovo* prior to emigration, is not known.

**Results:**

We have conducted single cell electroporations of a GFP-encoding plasmid into the dorsal midline of E2 chick NTs at the adrenomedullary level of the NC. Analysis of their derivatives, performed at E6, revealed that in most cases, labelled progeny was detected in both sympathetic ganglia and adrenal glands, where cells co-expressed characteristic marker combinations.

**Conclusions:**

Our results show that sympathetic neurons and adrenal chromaffin cells share a common progenitor in the NT. Together with previous findings we suggest that phenotypic diversification of these sublineages is likely to occur after delamination from the NT and prior to target encounter.

## Background

The neural crest (NC) is a transient structure in developing vertebrate embryos that arises at the interface between the epidermal and neural ectoderm. Upon neural tube (NT) formation, NC cells delaminate from its dorsal aspect and undergo an epithelial-to-mesenchymal transition followed by migration to their target locations. NC cells give rise to many different cell types, including neurons and glia of the autonomic and sensory nervous systems, neuroendocrine chromaffin cells in the adrenal medulla and other locations, thyroid C cells, melanocytes, and selected populations of mesenchymal cells [1]. The sympathoadrenal (SA) cell lineage is a major sub-lineage of the NC that gives rise to sympathetic neurons, intra- and extra-adrenal chromaffin cells, and the small intensely fluorescent (SIF) cells, which are intermediate between sympathetic neurons and chromaffin cells [2-5]. SA derivatives share several features, including the ability to synthesize, store, and release catecholamines, but also exhibit traits specific for each cell type, such as axons, dendrites, and neurofilaments, which are only found in sympathetic neurons but not chromaffin cells.

After delaminating from the NT, the earliest emigrating NC cells migrate ventrally to the area of the dorsal aorta [6-8]. Here, they acquire catecholaminergic features, instructed by bone morphogenetic proteins 2/4/7, which are secreted by cells of the wall of the dorsal aorta [9,10]. Furthermore, a series of interacting transcription factors including MASH-1, Phox2a, Phox2b, dHand, Gata2/3, and Insm1, were found to be essential for the generation of the SA catecholaminergic phenotype [11-19]; for reviews see [2-6,20,21].

Cell culture studies using isolated SA progenitor cells from embryonic and early postnatal sympathetic ganglia and adrenal gland have suggested that glucocorticoids play a crucial role in the diversification of SA cells into sympathetic neurons and chromaffin cells [22-24]; for reviews see [2,5,6,25], by suppressing a neuronal differentiation programme in SA cells destined to become chromaffin cells. However, analysis of mice deficient for the glucocorticoid receptor [26] or lacking an adrenal cortex [27] revealed that chromaffin cell differentiation was largely unimpaired suggesting that cues unrelated to glucocorticoid receptor signalling and the adrenal cortex triggered the chromaffin phenotype. This notion was corroborated and extended by studies showing that chick SA progenitors are already heterogeneous in terms of neurofilament-M (NF-M) expression prior to populating the adrenal gland and sympathetic ganglia [28] with presumptive neuronal cells co-expressing tyrosine hydroxylase (TH) and NF-M, and presumptive neuroendocrine cells being TH-positive and NF-M-negative. This indicated that specification of the respective phenotypes occurred either during migration or even prior to migration at the level of the NT.

A recent study by Krispin et al. [7,29] showed that in the trunk NT of avian embryos, precursors for sympathetic ganglia, Schwann cells, sensory dorsal root ganglionic neurons, and melanocytes leave the NT in successive, largely non-overlapping waves, some being already distinct while still residing in the NT. Although adrenal chromaffin cells were not addressed in this study, the results raised the possibility that sympathetic neurons and chromaffin cells might also already be distinct at the level of the NT. We therefore conducted single cell electroporations (EPs) of green fluorescent protein (GFP)-DNA into pre-migratory NC cells at the level of somites 18–24 (“adrenomedullary level”), where both chromaffin cells and sympathetic neurons arise [30]. The timing and location of EPs was directed to label the earliest progenitors that undergo delamination, previously shown to generate the sympathetic lineage [7,29], and the progeny of labelled cells was then analysed at E6 following homing to their target areas. Our results indicate that in more than 80% of the cases the progeny of a single labelled cell ends up in both sympathetic ganglia and adrenal gland, being mostly NF-M-negative in the adrenal gland and NF-M-positive in sympathetic ganglia. This suggests that chromaffin cells and sympathetic neurons share a common progenitor in the NT and that diversification of the phenotypes occurs during migration or assembly in primary sympathetic ganglia at the dorsal aorta.

## Results and discussion

### Transfection of GFP-encoding DNA into single dorsal NT progenitors

**Figure 1:**
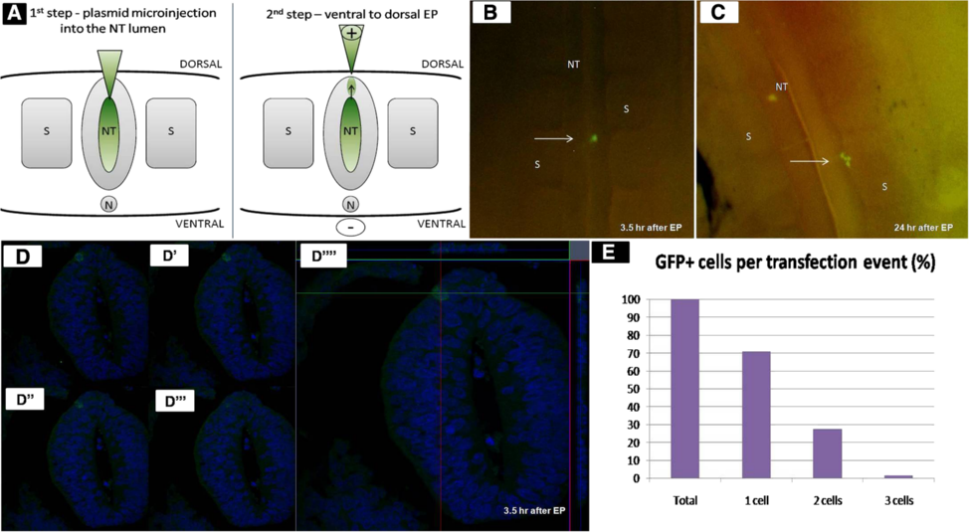
**Single-cell EP of GFP-DNA into the dorsal neural tube.** (**A**) Diagram showing the two-step procedure for single-cell EP, beginning by plasmid microinjection and followed by application of ventro-dorsally directed electric pulses. Note position of electrodes, injection site, and position of single electroporated cell. (**B,C**) Live-visualization *in ovo* at 3.5 h (**B**) and 24 h (**C**) after EP. (**D**) Histological cross section showing confocal analysis of a single GFP-expressing cell in the dorsal NT 3.5 h after EP visualized with an anti-GFP-antibody, combined with DAPI nuclear staining (blue). D, D’, D”, and D”’ show four representative focal planes out of 23 planes from a Z-stack of a 10 μm thick section. Samples were optically screened at 0.35 μm increments. D”” shows orthogonal projections. (**E**) Summarizes results of 69 experiments, with 71% successful single-cell-EPs verified by immunohistology, and 27.5% of the cases, where two cells were seen. In 1.5% of the cases, GFP was visualized in three cells. Scale bar: 10 μm. NT, neural tube; S, somites.

Stereomicroscopic and histological analyses were performed 3.5 h following focal EP of the dorsal NT to examine the extent of single cell labelling upon initial GFP protein expression. Figure 1A schematically reveals the position of electrodes, injection site of GFP-DNA, and a single labelled cell in the dorsal NT. A single GFP+ cell at 3.5 h is shown by live-epi-fluorescence imaging (Figure 1B). By 24 h the cell had migrated out from the NT and generated a cluster of three cells (Figure 1C). Further confirmation of clonal transfection was obtained by confocal analysis (Figure 1D-D””). A summary of evaluated cases (Figure 1E) reveals that in 49 out of 69 cases showing labelled cells, only one GFP+ neuroepithelial cell (71%) was detected at the NT level. In 19 cases (27.5%) two cells were detected, and in one more case three cells were visible. Notably, the number of embryos showing labelled cells was approximately 12% of total electroporations performed; this low efficiency is in further support of the clonal nature of transfections.

### Distribution of the progeny of single NC progenitors following target organ colonization

**Figure 2:**
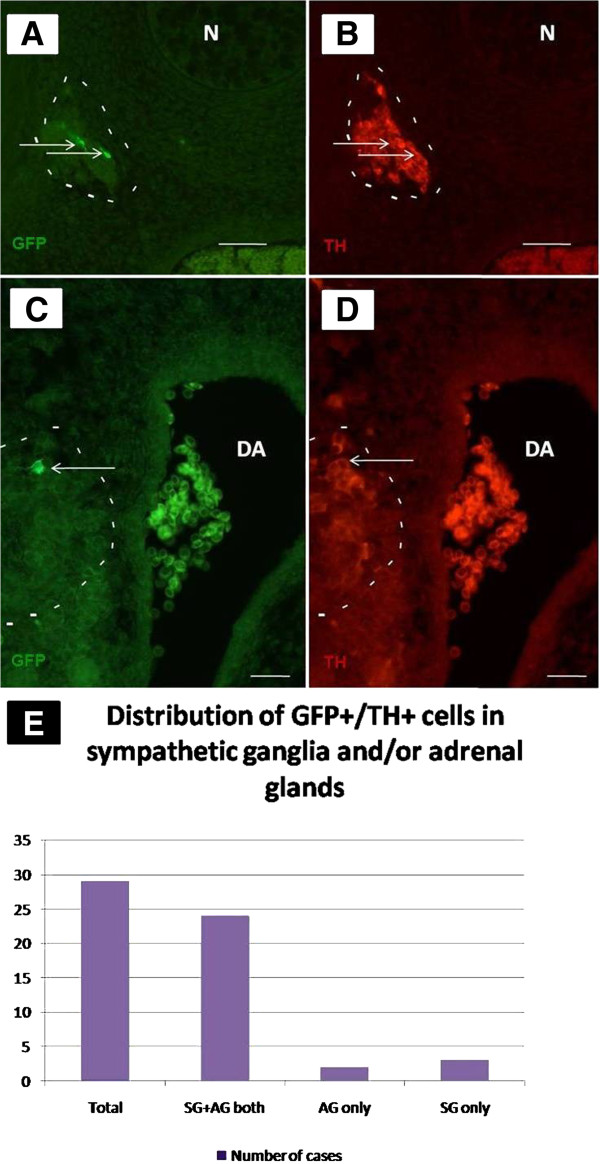
**Analysis of GFP-labelled cells in sympathetic ganglia (A,B) and adrenal gland (C,D) at E6.** (**A**) Sympathetic ganglion (white demarcation) harbours two GFP-positive cells (green). (**B**) TH antibody staining of the same section as in (A). Arrows mark the two co-labelled cells. (**C**) Adrenal gland (white demarcation) harbours one GFP-positive cell (green). (**D**) TH antibody staining of the same section as in (C). Arrows point to the TH+/GFP-positive cells. Note the autofluorescence of red blood cells in dorsal aorta (**C,D**). (**E**) Distribution of GFP+/TH+ cells in sympathetic ganglia and adrenal gland at E6, following single cell EP into the dorsal NT at E2. Scale bars: 50 μm. N, notochord; DA, dorsal aorta.

Electroporated embryos that showed a single labelled progenitor at 3.5 h post-EP were further incubated till E6 when adrenal gland and sympathetic ganglia were established and the progeny of electroporated cells had reached these organs. Embryos were then fixed, paraffin embedded and stained with GFP and TH antibodies. Figure 2 shows GFP+/TH+ cells in a sympathetic ganglion (Figure 2A,B) and adrenal gland (Figure 2C,D), respectively. As summarized in Figure 2E in 24 out of 29 cases of the single-cell EPs we detected GFP+/TH+ cells in both locations, i.e., adrenal gland and sympathetic ganglia (*P* = 0.0004). In two cases GFP+/TH+ cells were found within the adrenal gland only, and in another three cases in sympathetic ganglia only. The number of GFP-positive cells in each tissue varied from 1 to 18 cells in sympathetic ganglia, and 2 to 12 in adrenal glands, respectively (Table 1). Together, the number of cells in clones within sympathetic ganglia compared to adrenal glands was not statistically different (*P* = 0.5). Notably, in the 29 cases presented in which labelled progeny were detected in SA derivatives no additional NC derivatives were found to contain labelled cells. This confirms the existence of early fate restrictions as initially described by Krispin et al. [7,29] and more specifically, it further supports the notion that SA progenitors are segregated from the other neural derivatives of the NC.

**Table 1:** Number of GFP+ cells per clone in the various derivatives

**Embryo/Case ID**	**SG**	**AG**
# 1	3	4
# 2	3	3
# 3	6	6
# 4	5	5
# 5	2	2
# 6	2	3
# 7	7	3
# 8	6	2
# 9	5	6
# 10	7	2
# 11	2	3
# 12	10	12
# 13	3	3
# 14	2	3
# 15	11	10
# 16	6	3
# 17	4	3
# 18	3	2
# 19	5	5
# 20	1	3
# 21	5	5
# 22	18	10
# 23	6	5
# 24	4	10
# 25	6	0
# 26	8	0
# 27	6	0
# 28	0	4
# 29	0	5

Results represent the number of GFP+/TH+ cells per clone present in sympathetic ganglia (SG), adrenal gland (AG), or in both tissues counted in a total of 29 labelled clones.

**Figure 3:**
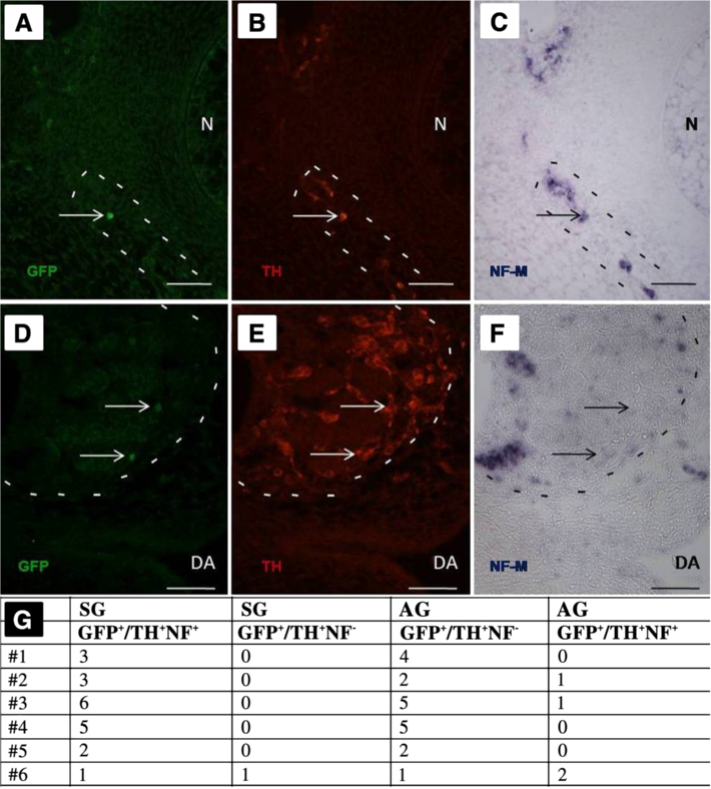
**Analysis of GFP-labelled cells derived from a single clone in a sympathetic ganglion (A-C) and adrenal gland (D-F) at E6 using antibodies to GFP (A,D), TH (B,E), and *in situ *hybridization for *NF-M *mRNA (C,F).** (**G**) Distribution of GFP+/TH+/*NF+* and GFP+/TH+/*NF-* cells in sympathetic ganglia (SG) and adrenal gland (AG) at E6. Scale bars: 50 μm. N, notochord: DA, dorsal aorta.

In mammalian and avian sympathetic ganglia, neurons are not the exclusive type of cell found within the ganglion. In chick, sympathetic ganglia harbour about 25% chromaffin-like cells [31]. Likewise, mammalian and avian adrenal glands contain a small proportion of neurons, in addition to the chromaffin cells [32,33]. Thus, the localization of a TH+ cell in sympathetic ganglia and adrenal glands, respectively, does not allow to unequivocally identify it as a neuron or chromaffin cell, respectively. However, in the chick embryo *NF-M* expression is indicative of a neuronal phenotype [28,34]. We therefore performed *NF-M in situ* hybridization in combination with GFP- and TH-immunostaining to verify neuronal and neuroendocrine chromaffin phenotypes in the respective locations. Figure 3A-C shows a GFP+/TH+/*NF-M*+ cell in a chick sympathetic ganglion at E6, confirming this cell as a neuron; Figure 3D-F reveals two GFP+/TH+/*NF-M*- cells in an E6 adrenal gland indicating that these are chromaffin cells. All labelled cells shown in Figure 3 are derivatives of the same clone. In total, 6 single-cell electroporated embryos (out of 24 embryos with derivatives detected in both sympathetic ganglia and adrenal gland) were analysed for GFP and TH immunoreactivities, and *NF-M* mRNA expression. Figure 3G shows that only one sympathetic ganglion contained, in addition to neurons, a GFP+/TH+/*NF-M*-negative, presumptive chromaffin-like cell, while three out of six analysed adrenal glands harboured neurons in addition to chromaffin cells. Hence, most cells (1–6) in sympathetic ganglia are *NF*-positive (*P* <0.001) and most cells (1–5) in adrenal glands are *NF*-negative (*P* = 0.0017).

Altogether, our data suggest that a single NC progenitor residing in the NT before delamination gives rise to both chromaffin cells and sympathetic neurons. Our findings therefore strongly support the notion that sympathetic neurons and chromaffin cells still share a common progenitor in the dorsal NT prior to delamination.

Consistent with this result, additional NC-derived sublineages are likely to be segregated only after emigration. For example, neurons and glia of sensory ganglia become segregated within the DRG themselves by Notch-dependent lateral inhibition [35]. A similar mechanism might account for segregation of SA progenitors as members of the Delta/Notch family are expressed in subsets of ventrally migrating SA cells [6].

Although growing evidence suggests that fate restrictions in the NC may occur prior to cell emigration [7,29,36-38], this notion should be systematically examined for specific derivatives by single cell lineage analysis *in vivo* at the appropriate time and axial location. In our study, this was done by performing single cell EP into the dorsal NT at the time and axial levels from which adrenal chromaffin and sympathetic neuronal progenitors delaminate. Single cell EP is an established method that allows the labelling of an individual cell in the midline of the NT before delamination. Although *in ovo* single cell EP is a technically demanding technique, with a success rate in our experiments of only 12%, we preferred EP over all other available methods, since it lacks the potential physical damage to the embryo produced by biolistic gene gun, potential toxic effects of viruses [39], and does not harm the morphology of the tissue [40]. Notably, in our experiment, a clear GFP signal was already visible 3.5 h after EP, likely before most labelled cells underwent mitosis. Consistently, in 71% of cases, only one cell in the dorsal NT was detected, while 27.5% of cases revealed two cells. In the latter case, either two cells were originally electroporated, or a single cell was labelled shortly before undergoing cell division.

## Conclusions

Taken together, our results suggest that sympathetic neuronal and chromaffin NC precursors are not pre-specified at the NT level favouring the option that specification may occur after delamination. Since neuronal and chromaffin progenitors can already be distinguished in their locations prior to populating the final target sites, sympathetic ganglia and adrenal gland, respectively, specification must occur during migration from the NT to the dorsal aorta or early in the peri-aortic area. Underlying mechanisms need to be investigated.

## Methods

### Embryos

Fertilized white Leghorn chicken (*Gallus gallus*) eggs were from commercial sources (Haas, France). Embryos were staged according to Hamburger and Hamilton [41].

### *In ovo* manipulations

An expression construct encoding an enhanced version of GFP, the pCAGGS-AFP (5 μg/μL) [42,43] was microinjected into the NT at the level of somites 18–24 of 18–24 somite-stage embryos (HH13). Care was taken to label cells opposite recently segmented epithelial somites as the dorsal midline area of the NT at this stage harbours progenitors that mostly generate sympathetic cells [7,29]. For focal EP of dorsal NT cells, a tungsten L-shape negative electrode was placed underneath the blastoderm. The positively charged electrode with the DNA-filled micropipette was inserted into the lumen of the NT. DNA at a concentration of 5 μg/μL was injected into the lumen and the micropipette was lifted up, until the tip of the micropipette touched the NT (Figure 1A, [7,29]). Immediately after injection four electrical pulses of current at a voltage of 60 V for 15 ms were applied at 100 ms intervals using an ECM® 830 electroporator (BTX Harvard Apparatus). Single cell labelling was achieved when the resistance of the glass micropipette was 1.8–2.2 MΩ (measured after the pulse) using a Multimeter (Alcron). Embryos were re-incubated for 3.5 h to monitor initial expression of GFP by inspection under a stereomicroscope with fluorescent attachment (Zeiss). Eggs were then re-incubated for an additional 20–24 h after EP to confirm that the cell had delaminated from the NT. Successfully electroporated embryos were then re-incubated until E6.

### Embryo processing and sectioning

Embryos were fixed in 4% formaldehyde overnight, washed twice in PBS, subsequently dehydrated in ascending concentrations of ethanol and acetone, and embedded in paraffin (Leica Biosystems). Paraffin blocks were cut into 10 μm thin sections on a microtome (Leica), and sections were mounted on Superfrost slides (Langenbrinck). Paraffin sections were deparaffinized and rehydrated before further processing.

### Immunohistochemistry and *in situ* hybridization

Deparaffinized sections were immunolabelled with antibodies for GFP and TH combined with *in situ* hybridization for NF-M. A non-radioactive *in situ* hybridization for chick-specific neurofilament-M (NF-M) was performed [44]. The riboprobes were diluted 1:10 in Tris/EDTA-buffer. Detection was performed with an anti-DIG antibody coupled to alkaline phosphatase and 4-nitroblue tetrazolium chloride/5-bromo-4-chloro-3-indolyl-phosphate (NBT/BCIP; Roche). Then, sections were processed for double-immunostaining with antibodies against TH and GFP. Before applying the primary antibody, antigen retrieval was performed to uncover the epitope of the TH antigen. Then, sections were incubated in blocking solution (0.5% Triton-100, 2% BSA, 1 x PBS) for 1 h. Next, they were incubated over night at 4°C with the primary antibodies to GFP (dilution 1:200; Molecular Probes-Invitrogen), and TH (dilution 1:400; Chemicon – Milipore). Secondary antibodies were: Cy3^TM^-568 goat anti-mouse (IgG) for detection of TH (dilution 1:400; Invitrogen). For visualization of GFP secondary Biotin-SP-conjugated Donkey anti-rabbit (IgG) antibody (dilution 1:100; Jackson ImmunoResearch) was applied for 1 h, then sections were washed with PBS. After washing, Cy2^TM^-conjugated streptavidine (dilution 1:200, Jackson ImmunoResearch) was applied for 1 h. Nuclei were counterstained with 4’,6’-diamidino-2-phenylindole dichloride (DAPI) (dilution 1:10,000 in 1xPBS, Boehringer). Sections were incubated for 5 min with DAPI, then washed in 1 x PBS and mounted with fluorescent-covering medium Fluoromount-G (Southern Biotech).

### Data analysis and statistics

The *in ovo*-images were taken with a Zeiss camera equipped with the AxioVision programme, and images were saved in JPG or TIFF format. If necessary, brightness and contrast were adjusted to the entire image using AxioVision and Photoshop 5.0 (AdobeSystems, USA), merged images were prepared using GIMP 2.0. Analysis of paraffin sections was performed using a Zeiss Axiophot microscope. Confocal scanning was performed on 11 embryos, including Z-stacks and orthogonal projections. A Zeiss confocal microscope with a 364 nm laser, ZEN 2010 software and x63 objective (1.2 W) was used. Within the NT, a single GFP-labelled cell was apparent in one transverse section.

To evaluate a common progenitor for chromaffin cells and sympathetic neurons, as well as neuronal (NF-M-positive) or neuroendocrine (NF-M-negative) cells, the χ^2^ test was used. Analysis of cell numbers in sympathetic ganglia *vs.* adrenal gland was performed using the *t*-test (*P* = 0.5).

## Abbreviations

AG: Adrenal gland; DAPI: 4’,6’-diamidino-2-phenylindole dichloride; E: Embryonic day; EP: Electroporation; GFP: Green fluorescent protein; HH: Hamburger/Hamilton; ISH: In-situ-hybridization; NC: Neural crest; NF: Neurofilament; NT: Neural tube; PBS: Phosphate-buffered saline; SA: Sympathoadrenal; SG: Sympathetic ganglion; TH: Tyrosine hydroxylase.

## Competing interests

The authors declare that they have no competing interests.

## Authors’ contributions

SS and MS participated in the design, the execution, and/or analysis of the majority of experiments. KH and SK helped with the interpretation of data and *in situ* hybridizations. CK and KU designed the study, interpreted the results, and wrote the manuscript with the help of SS. All authors read and approved the final version of the manuscript.

## Authors’ information

Chaya Kalcheim and Klaus Unsicker are joint senior authors.
